# Structure–Function Analysis of RBP7910: An Editosome Z-Binding Protein in Trypanosomatids

**DOI:** 10.3390/molecules28196963

**Published:** 2023-10-07

**Authors:** Curtis Ehlert, Naghmeh Poorinmohammad, Saba Mohammaei, Linhua Zhang, Reza Salavati

**Affiliations:** 1 Institute of Parasitology, McGill University, Montreal, QC H9X 3V9, Canada; curtis.ehlert@mail.mcgill.ca (C.E.); nagmeh.poorimohammad@mcgill.ca (N.P.); saba.mohammaei@ircm.qc.ca (S.M.); linhua.zhang@mcgill.ca (L.Z.); 2 Department of Biochemistry, McGill University, Montreal, QC H3A 1A3, Canada

**Keywords:** *Trypanosoma brucei*, editosome, z-binding proteins, microscale thermophoresis, protein structure prediction, molecular docking

## Abstract

RNA editing, a unique post-transcriptional modification, is observed in trypanosomatid parasites as a crucial procedure for the maturation of mitochondrial mRNAs. The editosome protein complex, involving multiple protein components, plays a key role in this process. In *Trypanosoma brucei*, a putative Z-DNA binding protein known as RBP7910 is associated with the editosome. However, the specific Z-DNA/Z-RNA binding activity and the interacting interface of RBP7910 have yet to be determined. In this study, we conducted a comparative analysis of the binding behavior of RBP7910 with different potential ligands using microscale thermophoresis (MST). Additionally, we generated a 3D model of the protein, revealing potential Z-α and Z-β nucleic acid-binding domains of RBP7910. RBP7910 belongs to the winged-helix–turn–helix (HTH) superfamily of proteins with an α_1_α_2_α_3_β_1_β_2_ topology. Finally, using docking techniques, potential interacting surface regions of RBP7910 with notable oligonucleotide ligands were identified. Our findings indicate that RBP7910 exhibits a notable affinity for (CG)n Z-DNA, both in single-stranded and double-stranded forms. Moreover, we observed a broader interacting interface across its Z-α domain when bound to Z-DNA/Z-RNA compared to when bound to non-Z-form nucleic acid ligands.

## 1. Introduction

*Trypanosoma brucei* employs an essential post-transcriptional RNA editing process which involves uridine insertion/deletion to generate functional mitochondrial mRNAs for the oxidative phosphorylation system. Specifically, a multiprotein complex called the editosome is responsible for this process. The editosome encompasses distinct subcomplexes with specialized functions. The RNA editing core complex (RECC) orchestrates the enzymatic phases of RNA editing. Concurrently, the RNA editing substrate-binding complex (RESC) establishes the structural basis for interactions between guide RNAs (gRNAs) and targets mRNA sequences. Additionally, the RNA helicase complexes (REH2C) demonstrate ATP-dependent 3′-5′ dsRNA-unwinding activity [1,2,3].

RBP7910 is a member of RESC and is reported to interact with other editosome proteins in an RNA-dependent manner. The protein is the first identified trypanosome protein with Z-DNA-binding domains, possessing two: one located in its N-terminus, known as Z-α, and another in its C-terminus, known as Z-β, having the potential to interact with the Z-conformation (left-handed) of nucleic acids [4]. Consequently, RBP7910 exhibits homology to Z-DNA-binding proteins (ZBPs) [5]. Enzymes such as RNA polymerase and helicases responsible for producing underwound double-stranded RNA and DNA can induce the Z-conformation in nucleic acids, recruiting protein machinery after formation [6]. This structure exhibits a left-handed orientation and features a zig-zag pattern, frequently found in purine–pyrimidine rich repeats, denoted as (C.G.) in [7].

The Z-DNA binding motif adopts the structure of the known winged-helix–turn–helix motif [5]. ZBPs possessing a Z-α domain are classified within the Z-α family (Pfam ID: PF02295) and the broader winged-helix superfamily. This superfamily, in turn, is a member of the helix–turn–helix clan (Pfam ID: CL0123). The simplest secondary structure of this motif comprises a core of three helical bundles and two beta-strands in the wing part (α1α2α3β1β2). This domain has evolved to include extended parts such as an extra beta-strand between the α-helix 1 and α-helix 2 (α1β1α2α3β2β3), which is the most common topology for ZBPs [8].

In ZBPs, the key interacting regions within the Z-α domain are the α-helix 3 and the wing region, consisting of two β-sheets. A multiple-sequence alignment analysis revealed high conservation in the N-terminus of the RBP7910 protein among trypanosomatida and other ZBPs with a Z-α domain, including human ADAR1 (double-stranded RNA-specific adenosine deaminase) [4]. ADAR1 is responsible for RNA editing in humans, converting adenosine into inosine in coding mRNAs originating from the nucleus [5]. This process involves ADAR1 binding to the dsRNA through its DSRM motif, and to the Z-forming RNA region via its Z-α domain, leading to editing within an extension of 150 base pairs on either side of this region, illustrating the Z-α domain’s capability to bind to both Z-DNA and Z-RNA [5]. Conversely, the Z-β domain shows low conservation in RBP7910. Generally the Z-β domain lacks a well-defined functional role, although exceptions exist, such as the Z-β domain of human ZBP1, known for its DNA-binding function [9]. Understanding the role of the Z-β domain in human ADAR1 is an ongoing area of research, with a current hypothesis suggesting a potential role in metal binding, possibly contributing to protein dimerization for the stabilization of RNA or DNA duplexes [9].

While Zα is known to bind Z-DNA or RNA, the Z-α domain of human ADAR1 can also bind the parallel-stranded G-quadruplex (G4) structure formed in the MYC promoter region [10]. To form a G-quadruplex, one generally requires stretches of DNA with a repeated pattern of guanines, often denoted as G-rich sequences. Interestingly, a study conducted by Leeder et al. [11] shows G4 formation in the G-rich pre-edited mitochondrial mRNA of specific trypanosomatid parasites. Through bioinformatic and in vitro analyses, they identified up to 27 G4s in pre-edited RNA. They present a model that during transcription, the editosome unwinds half of these G4s, aiding in the creation of an intermediate pre-edited RNA-gRNA. After this intermediate state is achieved, it can proceed through RNA editing.

Given these considerations, this study exclusively focuses on the Z-α domain of RBP7910. Using an electrophoretic mobility shift assay (EMSA), it was demonstrated that RBP7910 exhibits a high affinity for RNA sequences rich in AU or U nucleotides [4]. However, it remained imperative to ascertain whether or not this protein genuinely functions as a Z-binding protein. To address this, a microscale thermophoresis (MST) test was performed to investigate the interacting interface and assess the binding affinity of RBP7910 for different oligonucleotide ligands. This technique is used to study interactions between biomolecules via the measurement of changes in fluorescence intensity as a result of temperature gradients induced by the use of an infrared laser. MST is valuable for determining binding affinities and characterizing molecular interactions in various biological systems [12]. Additionally, molecular docking analysis was carried out to corroborate MST findings and identify potential interacting regions with various types of ligands.

## 2. Results and Discussion

### 2.1. Binding Affinity of RBP7910 with Potential Oligonucleotide Ligands

A summary of K_d_ and confidence values for all oligonucleotides mentioned in the Materials and Methods section, tested against RBP7910, can be seen in Table 1.

Based on the MST results, the lowest recorded K_d_ value is observed for the d(GC)6 ss while the values are much higher for r(UA)6 and r(GC)6 oligonucleotides. Conversely, r(UUUU)3, r(AUUU)4, and r(AUUC)3 ligands all returned largely consistent K_d_ scores across single-stranded and double-stranded forms. Accordingly, the estimation of the binding affinity between the RBP7910 protein and various oligonucleotides using MST generally revealed that RBP7910 exhibited the highest affinity for single-strand d(GC)6, featuring purine–pyrimidine repeats likely of adopting a Z-form conformation in solution. In contrast, both r(UA)6 ss and r(GC)6 ss, also containing purine–pyrimidine repeats, showed higher K_d_ values compared to r(UUUU)3 ss, r(AUUU)4 ss, and r(AUUC)3 ss oligonucleotides, which are unlikely to form Z-RNA structures in solution. d(GC)6 ds also reported the lowest K_d_ value among double-stranded oligonucleotides—while r(GC)6 ds reported the highest K_d_ value for double-stranded ligands, r(UA)6 ds reported the third lowest, behind only r(AUUU)4 ds. Previous studies have highlighted RBP7910’s strong binding affinity for r(UUUU)3 and r(UA)6 sequences, despite the ZBD conformation of the RBP7910 Z-α domain [4]. Given this, the high binding affinity demonstrated here by single-stranded and double-stranded d(GC)6 oligonucleotides is noteworthy.

### 2.2. Three-Dimensional Structure Prediction of RBP7910 

**Figure 1:**
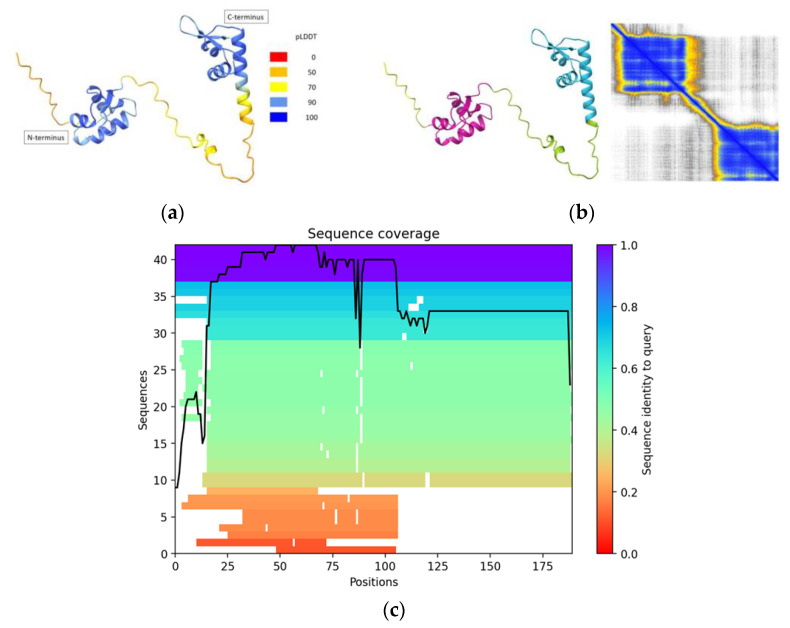
AlphaFold2 results to model RBP7910 structure. (**a**) pLDDT map showing a high degree of predicted accuracy within Z-α domain, but less in Z-β domain, specifically its first α-helix. Random coil linker region demonstrates low-moderate structural confidence; (**b**) PAE demonstrating high confidence within putative Z-α, Z-β domains, but uncertain relative positions between the two; (**c**) MSA chart reporting MMseqs2-determined values showing high sequence identity to query among ~40 sequences in the RBP7910 N-terminus Z-α region. Sequence number and identity to query are decreased for the C-terminus Z-β region.

The model revealed two distinct ZBDs as observed in Figure 1: one located at the protein’s N-terminus (tentatively identified as the Z-α domain) and the other positioned at its C-terminus (tentatively identified as the Z-β domain). The AlphaFold2 prediction for the full-length RBP7910 structure demonstrates high confidence scores particularly within the Z domains, with pLDDT values exceeding 90 for most parts of these domains (see Figure 1a). Notably, pLDDT scores exceeding this threshold are known to indicate not only accurate backbone predictions but also correctly oriented side chains, with a reported accuracy of approximately 80% for χ1 rotamers in recent PDB test data sets [13]. This underscores the high quality and reliability of our model which makes it well-suited for applications such as characterizing binding sites.

Structurally, the Z-α domain was anticipated to feature the arrangement α1α2α3β1β2, a recognized domain commonly associated with DNA binding (Figure 1). The Z-β domain located at the C-terminus also exhibited characteristics of a DNA-binding domain, adopting a secondary structure of α1α2α3α4β1β2. Notably, a brief α-helix 3 (spanning Gln160 to Tyr162) was observed, with only one residue (Gly163) separating it from α-helix 4. Generally, the pLDDT score returned a somewhat lower confidence level for the potential Z-β domain, particularly in its initial α-helix.

Concerning the secondary structure prediction of the RBP7910 protein, the results returned the canonical structure of the winged-helix motif featuring three helical bundles and two strands in the wing region (α1α2α3β1β2). Interestingly, the winged-helix motif has evolved into other architectures with similar folds. For example, this motif may also have a third β-strand between α-helix 1 and 2, compacted against two other β-strands in the wing part. This three-stranded version of the winged- helix motif can be observed in selecting ZBPs as α1β1α2α3β2β3, with an extended β-strand 1 which does not commonly appear in ZBP PDB crystal structures. For instance, 3f22, 2gxb, 3irq and 3irr, PDB IDs for crystal structures of the human ADAR1 Z-α domain, do not have the predicted β1-strand in their structures, although 1qbj does. This implies that the β1-strand in the ZBPs can be inconsistent among their 3D-structures, which may also apply to the Z-α domain of RBP7910. Crystallographic validation would be required to validate the predicted structure of this domain.

Beyond these domains, the structure of RBP7910 featured random coil regions, indicating lower structural confidence or the presence of intrinsically disordered regions (IDRs). IDRs can provide flexibility to the protein structure, allowing it to adapt to different conformations of Z-DNA [14]. This flexibility is essential for the protein to effectively interact with the dynamic and less structured Z-DNA molecule. On the other hand, it is reported that in some cases, IDRs may only become structured on the binding Z-DNA or Z-RNA, which represents a possible important role of IDRs in the tuning of interactions with Z-DNA and Z-RNA [15]. The predicted aligned error (PAE) provided insights into the structural confidence within the Z-α and Z-β domains, but not between them. It is presumed that the domains would have relative freedom of movement due to the presence of a random coil linking region.

In addition to generating a 3D structure, a multiple-sequence alignment (MSA) graph was generated for the entire RBP7910 protein using MMseqs2 to gauge the primary structure similarity across sequences (Figure 1c). This resulted in ~40 sequences being found to match the RBP7910 input sequence at near a 1.0 sequence identity to query in the Z-α domain of the N-terminus region (amino acids 20–81). The number of sequences covering the C-terminus Z-β domain (amino acids 120–189) is noticeably lower, though consistent. This implies greater conservation among known sequences for the RBP7910 N-terminus.

Once the AlphaFold2-modeled RBP7910 structure was attained, structural similarity queries were launched for both the Z-α and Z-β domain using RUPEE. When querying the Z-α domain, each PDB chain ID within the top 30 structures was found to originate from a ZBP, with PKZ (PDB: 4lb5B), a fish-specific protein kinase associated with cell apoptosis, being the protein with the highest chain geometric similarity. Other notable ZBPs include E3L (PDB: 7c0iA), a protein produced by the vaccinia virus, and ADAR1 (PDB: 2gxbB). When querying the C-terminus Z-β domain, PKZ was heavily featured as a top-scoring protein across various PDB IDs. DCN1-like proteins, non-ZBPs, were recurring top-scoring models as well. Generally, RMSD values and TM-scores were lower for the homology results of the Z-β domain compared to those for the Z-α domain. This denotes a more conserved function or binding mechanism in the Z-α domain across different proteins. In contrast, the higher variability in the Z- β domain may indicate a wider range of functions or binding specificities associated with proteins in this region, which needs further experimental analysis.

### 2.3. Prediction of the Binding Interface via Molecular Docking

Molecular docking was performed with HDOCK, using integrated protein–nucleic acid docking to map RBP7910 (receptor) interactions for a ligand molecule input. To sample binding modes across the entire protein, global docking was performed, and interacting residues were recorded for the top five docking models per ligand. To this end, the entire predicted RBP7910 structure was selected as a receptor against various oligonucleotide ligands used in the MST study (single-stranded and double-stranded), including the non-Z-form r(UUUU)3, r(AUUU)4, r(AUUC)3, and Z-form d(GC)6, r(GC)6, and r(UA)6 sequences. A summary of ligand sequences and applicable docking score standard deviation values can be found in Table 2. The recorded standard deviation (SD) values are largely similar, though generally higher for Z-form nucleic acids, particularly d(GC)6 ds. Interestingly, this does not apply to r(GC)6 ds and r(UA)6 ds ligands, which returned the smallest standard deviation values.

It is important to note that SD is used as a ranking metric instead of the docking score as the docking score provided by HDOCK does not directly correlate with binding affinity. This is particularly relevant in scenarios where the scoring function may have limitations or is not explicitly calibrated to represent thermodynamic binding constants. By employing SD, we assess the variability in docking poses generated during the simulations. This approach allows us to identify docking solutions that consistently demonstrate favorable interactions between the ligand and receptor, indicating robust binding potential. Moreover, SD provides a measure of the stability and reliability of the predicted binding modes. Therefore, in the absence of a direct binding affinity estimation from the HDOCK scoring function, the use of SD serves as a valuable means to discern and prioritize the most plausible docking solutions for further analysis.

**Figure 2:**
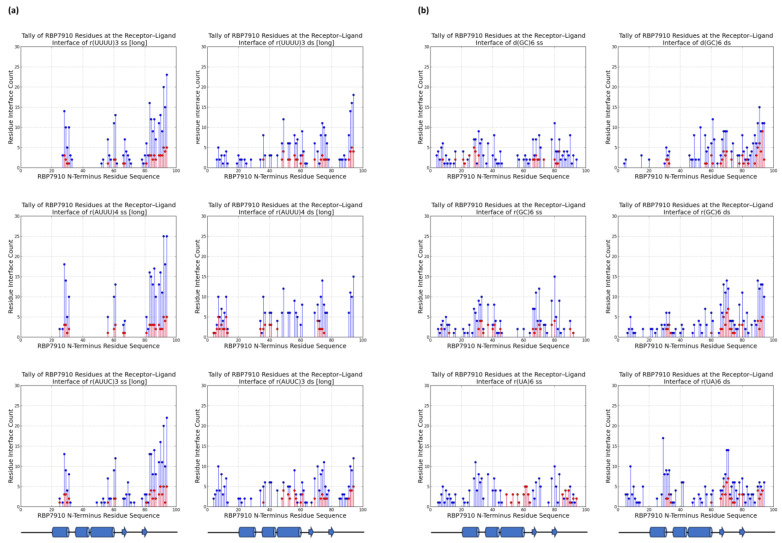
RBP7910 interaction sites from top 5 docking models (**a**) for non-Z oligonucleotides; (**b**) for Z-form oligonucleotides. For (**a**) or (**b**): single-stranded (**left**) and double-stranded (**right**). Blue data points summarize interactions from all 5 docking models, while interactions from the top docking model are superimposed in red. Results from top docking model are superimposed on data in red. The applicable RBP7910 region (amino acids 1–100) is defined by linear projection of the protein N-terminus and random coil domain.

For each ligand, a tally of RBP7910 residues at the receptor–ligand interface for the top five docking models was recorded to understand the possible binding regions of RBP7910in Figure 2. Residue H-bonds found along the protein were also tallied and graphed. In all cases, returned RBP7910 models were defined by amino acids 1–94, encompassing the N-terminus Z-α domain and random coil region.

**Figure 3:**
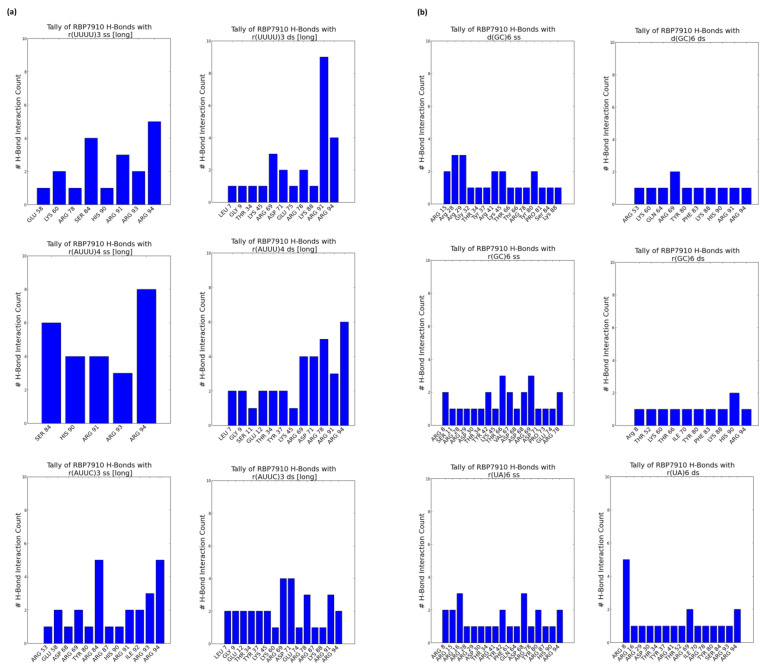
RBP7910 hydrogen bond sites from top 5 docking models (**a**) for non-Z oligonucleotides; (**b**) for Z-form oligonucleotides. For (**a**) or (**b**): single-stranded (**left**) and double-stranded (**right**). Data gathered from ChimeraX (version 1.7.dev202308312248).

Figure 2a summarizes these tallies across single-stranded and double-stranded non-Z oligonucleotide ligands. Blue data points summarize interactions from the top 5 docking models without bias to the docking score, while superimposed red data points summarize interactions from the top docking model alone. In the former case, interactions were most common between the β-strand 2 and random coil RBP7910 region until residue Arg94, and while α-helix 1 (amino acids 20–29) and α-helix 3 (amino acids 45–60) are observable interaction sites, these are less pronounced than those of the random coil region. H-bonds were predominantly found between the β-strand 2 and random coil regions (amino acids 81–94), where the residue interface count is the densest (Figure 3a). Thich could be attributed to the heavy presence of arginine residues in the region (Arg78, Arg87, Arg91, Arg93, and Arg94), serving as excellent H-bond donors due to their guanidinium moiety. These binding sites extended into α-helix 3 and the wing region (amino acids 61–81) for r(UUUU)3 ss and r(AUUC)3 ss ligands.

Double-stranded non-Z oligonucleotides exhibit a broader sampling of the RBP7910 N-terminus, particularly for residues preceding α-helix 1 and a greater number of α-helix 3 and wing region residues. Much like single-stranded non-Z ligands, the region with the most frequent interaction site is observed as the random coil region up to and including Arg94. This broader sampling of the protein interface is reflected in the residue H-bond tally, recorded as early as Leu7 and extending to Arg94 across all relevant ligands.

When docked against Z-form oligonucleotides (ss), RBP7910 interaction hits span a broader range of residues across the Z-α domain but exhibit less site-specificity when compared to that of non-Z ligands (Figure 2b). Dense interaction sites preceding α-helix 1 and within α-helix 2 (amino acids 35–43) and the wing region in particular are more common for single-stranded Z-form ligands, while interactions are significantly less frequent in the random coil region as residues approach Arg94, and instead predominantly center Tyr80 of β-strand 2. Recorded H-bond protein interaction regions within α-helix 1, α-helix 2, and the wing region are much more pronounced against single-stranded Z-form ligands compared to non-Z ligands, though fewer H-bonds are observed within α-helix 3 (Figure 3b).

When docked against double-stranded Z-form ligands, RBP7910 interactions are observably more prevalent within the random coil region until Arg94 (most notably for the d(GC)6 ds ligand) compared to when docked against single-stranded Z-form ligands nucleic acids; furthermore, dense and site-specific interaction sites are more commonly observed in the wing region between β-strands 1 and 2. H-bond interactions do not exhibit strong site specificity and are observed over a broad range of residues when docked against the r(UA)6 ds ligand; however, these sites are more prevalent in the RBP7910 random coil region when docked against d(GC)6 ds and r(GC)6 ds ligands.

According to our unpublished MST test data, key residues of RBP7910 show the lowest binding affinity towards r(UUUU)3 ss and r(AUUU)4 ss oligonucleotides when in vitro alanine point mutations are introduced at Arg53 and Thr52. As revealed in an extensive sequence conservation analysis of RBP7910 conducted by Nikpour et al., these two residues exhibited conservation within the Trypanosoma genus [4]. Furthermore, with a simple superimposition of the α-helix 3 structure of more distantly related Z-DNA binding proteins homologs such as Z-β human Z-binding protein 1 (PDB: 3eyi), Z-α Danio rerio PKZ (Zebra fish) (PDB: 4lb5), and Z-α human ADAR1 (PDB: 2gxb), these residues are conspicuously conserved. However, both Arg53 and Thr52 are infrequently identified as residue hits in docking results, most notably for single-stranded ligands—when tallying interacting residues across the top five binding models against r(UUUU)3 ss, Arg53 was counted twice and Thr52 once. Therefore, it is possible that instead of directly participating in ligand binding, these residues may stabilize protein conformation or contribute to its allosteric effects. It is also possible that Arg53 and Thr52 stabilize the protein–ligand complex via water-mediated interactions, a significant contributor to binding effects but which often goes ignored in in silico studies. To obtain a comprehensive understanding of the roles of Arg53 and Thr52, further in vitro experimental testing will be necessary. Furthermore, given that these residues reside in regions of the protein characterized by the highest pLDDT scores—a metric akin to the B-factor in conventional structural models—they are anticipated to demonstrate enhanced structural stability. This observation designates them as promising subjects for further investigation.

Moreover, though K_d_ values from MST tests demonstrate stark differences in RBP7910 binding affinity between Z-DNA and Z-RNA ligands, this is not well reflected in the docking results, which show little variation in interaction regions between d(GC)6, r(GC)6, and r(UA)6 oligonucleotides. While these docking results summarize interaction trends across the RBP7910 interface, further work must be carried out to pinpoint key residues and their impact on protein binding. While further docking tests can be performed to further isolate regions of interest, a more in-depth characterization of RBP7910-interacting residues would require in vitro mutagenesis tests against these ligands.

In general, the docking results showed that residues Tyr80–Arg94 of the random coil region (extending past the Z-α domain as defined by its α1α2α3β1β2 topology) were prominent interaction sites against single-stranded non-Z ligands, featuring both the highest residue tallies and most densely concentrated quantity of interaction sites. As mentioned earlier, this is likely attributed to the substantial presence of arginine residues Arg87, Arg91, Arg93, and Arg94, creating a dense environment extremely conducive to the generation of H-bonds. Interestingly, little interaction bias with this region was recorded for RBP7910 docking against single-stranded Z-form ligands, favoring sampling over a broader region of the RBP7910 N-terminus Z-α domain. It should be noted that global docking was performed in all cases, with no defined interaction regions of bias. This is consistent with known Z-DNA-contacting residues of ZBPs such as ADAR1, which are most commonly found in α-helix 3 and the beta hairpin β1-β2 wing region (15).

## 3. Materials and Methods

### 3.1. Protein Expression and Purification

RBP7910 was produced through expression in *E. coli*, followed by purification and the subsequent estimation of its concentration. GenScript undertook the cloning of RBP7910 constructs utilizing the PET-30a(+) vector, which featured a TEV cleavage site and a 6X-His-tag positioned at the N-terminal of the RBP7910 sequence. Subsequently, plasmid constructs were introduced into T7 Express lysY BL21 *E. coli* competent cells (high efficiency). These bacterial cells were then cultivated on L.B. plates supplemented with Kanamycin antibiotic, and incubated overnight at 37 °C. Single colonies from these plates were utilized to inoculate 25 mL of L.B./Kan pre-cultures, followed by an overnight incubation at 37 °C. To establish a 1 L LB/Kan cell culture, 18 mL of the pre-cultures were employed to inoculate the 1 L cultures, aiming for a starting OD600 of 0.05–0.1. The cultures were then incubated (at 37 °C, with 200 rpm shaking) for 1.5–2 h until the OD600 reached 0.4–0.6. At this point, IPTG was introduced to the cultures to achieve a final concentration of 0.5–1 mM. The cultures were further incubated at two distinct temperatures (37 °C and 30 °C), and cell harvesting was conducted after 1.5 h and 3 h of induction to evaluate the optimal conditions for protein expression. The cell cultures were centrifuged at 4 °C and 6000 rpm for 20 min. Subsequently, cell pellets were resuspended in 1X PBS, followed by another round of centrifugation at 4 °C and 10,000 rpm for 20 min. Finally, cell pellets were stored at −80 °C.

The cells were lysed using 3–5 mL of lysis buffer per gram of cell pellet. Following lysis, the cell lysate underwent sonication for a duration of 2 min (at 40 W, with a cycle of 15 s on and 30 s off), and samples were extracted to represent the total cell lysate. Subsequently, tubes containing the cell lysate were subjected to centrifugation at 4 °C and 10,000 rpm for 20 min, resulting in the collection of the supernatant. Samples were taken from both the supernatant and the total cell lysate and were combined with 2X SDS dye for subsequent analysis using Coomassie gel electrophoresis. The protein samples were then separated utilizing 15% SDS-PAGE gel to assess the level of expression.

Ni-NTA Agarose beads were utilized for the purification process. Initially, the sample supernatants were introduced into the columns, followed by one-hour incubation at 4 °C on a mixer. Subsequently, the flow-through was gathered and subjected to an additional pass through the column, followed by three washes using a wash buffer containing 50 mM imidazole. Then, elution buffer with 500 mM imidazole was introduced to collect eluates. This elution step was repeated three to four times with 30 min of incubation at 4 °C for each cycle. Following elution, dialysis was carried out using a buffer comprising 1% PBS and 10% glycerol, with samples undergoing overnight incubation at 4 °C. Finally, the samples were collected, concentrated, snap-frozen, and subsequently stored at –80 °C.

### 3.2. Estimating the Binding Affinity of RBP7910 with Different Ligands

In a prior investigation conducted by our research group, it was determined through an electrophoretic mobility shift assay (EMSA) that RBP7910 exhibits a heightened binding affinity towards r(UUUU)3 and r(UA)6 sequences [4]. Consequently, MST experiments employed AU-rich and U-rich oligonucleotides, specifically r(UUUU)3, r(AUUU)4, r(AUUC)3, and r(UA)6. Additionally, (CG)n DNA/RNA sequences were employed to comprehensively assess ligand binding affinity. The details of all utilized oligonucleotide sequences can be found in Table 3.

To find the potential ligand(s) for RBP7910, the binding affinity of this protein was estimated using MST [12]. Oligonucleotide sequences, procured from IDT, were fluorescently labeled with Cy5 TM at their 3’ terminus and purified via RNase-free HPLC by IDT. MST experiments were conducted using the Monolith NT.115 Blue/Red instrument from NanoTemper^®^ (München, Germany). The initial RNA concentration was 100 nM, with an assay concentration of 50 nM. Protein stocks were prepared at concentrations of 2 uM, 5 uM, or 8 uM, and assay concentrations were half of the stock values (1 uM, 2.5 uM, and 4 uM, respectively). Dilutions were made using RNase-free water, and RBB50 buffer (HEPES at 20 mM, pH 7.6, KCl at 150 mM, MgCl_2_ at 5 mM, BSA at 100μg/mL, and glycerol at 10%) served as the binding buffer. Prior to the MST experiment, oligos were denatured via heating in 95 °C water for 3 min. The water was then gradually cooled to 75 °C, and once it reached 25 °C, the oligonucleotides were ready for use. The first step involved pretesting the labeled oligos, followed by a binding check for the protein and ligands to determine complex formation. Upon achieving a satisfactory signal-to-noise ratio for binding, K_d_ values were calculated. The K_d_ confidence intervals were determined based on the variance of the parameter fitted using the widely adopted Levenberg–Marquardt algorithm for curve-fitting. The reported values correspond to the mean K_d_ value and its range within a 68% confidence interval. This process involved creating a 1:2 serial dilution series of the protein using binding buffer (RBB50) (with 16 dilutions), followed by the addition of a constant oligo concentration to the tubes. After mixing the protein and oligos, the tubes were incubated at room temperature for 30 min to 1 h to allow complex formation. Finally, reactions were loaded into capillaries and analyzed using the MST machine.

### 3.3. Modeling the 3D Structure of RBP7910

AlphaFold2 was implemented via ColabFold to model the 3D structure of the full-length protein sequence of RBP7910 (Tritryp ID: Tb927.10.7910). AlphaFold2 is widely cited for its high structural prediction accuracy compared to that of other available tools, accomplishing this through the use of neural networks to predict the 3D coordinates of each protein residue using feature extraction and iterative refinement [16]. It also returns data on structural predicted local distance difference tests (pLDDT) and predicted aligned errors (PAE), helpful frameworks for assessing the quality of output structures and their suitability for docking applications.

The resulting structure was then queried against the RUPEE webserver [17] in order to perform an structural similarity search. This webserver employs a rapid Needleman–Wunsch algorithm for geometric protein structure searches, enabling the assessment of structural resemblance within the Z-α and Z-β domains of RBP7910 [18]. This approach has previously been employed for querying Z-DNA binding proteins for structural similarities [19].

### 3.4. Molecular Docking

Docking was performed via the HDOCK web server [20], using the entire RBP7910 protein against oligonucleotides administered in the MST study to observe potential regions of interaction between Z- and non-Z-form nucleic acids. The entire RBP7910 structure as predicted via AlphaFold2 was selected as the receptor input, while various oligonucleotide constructs (Table 3) were selected as ligand inputs. HDOCK interacting region data were visualized using Python (v2. 7. 11), while the hydrogen bonds (H-bonds) present in models were identified using ChimeraX (version 1.7.dev202308312248) [21].

The buffer used in MST test reactions comprised HEPES (20 mM), allowing for greater pH stability, and reducing the likelihood of oligonucleotide secondary structure formation, as well as that of KCl, providing ionic strength to shield the oligonucleotides’ charges and minimize electrostatic interactions between them. Accordingly, idealized A-form single-stranded (ss) non-Z-RNA oligonucleotides (r(UUUU)3, r(AUUU)4, and r(AUUC)3) were created manually using Discovery Studio Visualizer (DSV) (v21.1.0.20298) [22]. Double-stranded (ds) non-Z-RNA oligonucleotides were generated by uploading the raw sequence into the HDOCK RNA/DNA 3D structure modeling tool, which for ds RNA/DNA inputs constructs a 3D structure using X3DNA, an integrated tool in HDOCK (http://hdock.phys.hust.edu.cn/ accessed on 12 September 2023), and a 5000-step AMBER59 MD minimization refinement [23]. Z-form oligonucleotides (d(GC)6, r(GC)6, and r(UA)6) were copied from a ds DNA GC-repeat oligonucleotide featuring a BZ junction (PDB ID: 5zuo), edited manually in DSV to include relevant non-binding site nucleotides towards the ligand 3′ end. These sites were further edited from the B-helix to A-helix when appropriate, accompanied with the modification of deoxyribose to ribose sugars for the entire structure. Due to the high degree of similarity between Z-DNA and Z-RNA helical parameters, the structural integrity of the RNA oligonucleotide binding site was believed to be largely unaffected by this modification. Once docking models were generated, a tally of RBP7910 residues in receptor–ligand interface residue pairs (<5 Å distance) was mapped above a linear projection of the N-terminus to understand protein interacting regiFigureons across ligand types for their respective top five docking models. Similarly, H-bonds along the protein were tallied and graphed. While the HDOCK scoring function is useful as it compares different binding models for the same receptor and ligand, it is not calibrated to experimental binding data, and therefore cannot be used to compare binding affinities between ligands [20]. Hence, only the standard deviation of the top five docking scores per ligand were documented, excluding the raw docking scores.

## 4. Conclusions

In conclusion, this study has illuminated the RBP7910 protein’s binding affinity with a spectrum of oligonucleotide ligands. The MST results underscored distinct preferences, with the highest affinity observed for single-stranded d(GC)6, potentially due to its purine–pyrimidine repeats favoring a Z-form conformation.

Intramolecular G4s form when a single G-rich DNA or RNA strand folds into a G4, commonly known as monomeric since it derives from one nucleic acid strand. In contrast, intermolecular G4s develop when multiple G-rich strands, which can be DNA, RNA, or hybrids of both, combined to form the G-quadruplex, either as dimers from two strands or tetramers from four strands. Consistent with Leeder’s model, RBP7910 may be recruited to the Z-conformation site because of a helicase or polymerase activity [11]. Next, the other factors may be recruited to initiate RNA editing. Interestingly, proteomics studies have shown the ~3-fold upregulation of RBP7910 protein at 4 h, 6 h, 12 h, 24 h, and 48 h based on quantitative proteomics during parasite differentiation [24]. Further research will result in a model framework substantially advancing our understanding of the dynamic interactions of RESC proteins involving a Z-RNA binding protein and contribute to the long-term goal of determining the stage-specific regulation of mt RNA processing in trypanosomatids.

The structural analysis further revealed potential Z-binding domains in both the N-terminal and C-terminal regions, with the former demonstrating higher conservation. These findings provide invaluable insights into the structural determinants governing RBP7910’s interactions with nucleic acids. While computational docking simulations illuminated potential interaction sites within RBP7910, experimental validation will be crucial to confirm and extend these findings. Notably, residues like Arg53 and Thr52, initially presumed to play direct roles in ligand binding, may instead contribute to structural stabilization or allosteric effects. Further in vitro studies are warranted to unravel the precise functions of these key residues. Overall, this study represents a significant stride towards comprehending the nuanced mechanisms underlying RBP7910’s recognition of various nucleic acid sequences, paving the way for future investigations into its biological significance.

## Figures and Tables

**Table 1:** K_d_ values measured by MST for RBP7910 interacting with different oligonucleotide ligands.

Ligands *	K_d_ (nM)	K_d_ Confidence **
r(UUUU)3 ss	147.31	19.65
r(UUUU)3 ds	1714.05	203.31
r(AUUU)4 ss	179.74	15
r(AUUU)4 ds	1884.56	419.35
r(AUUC)3 ss	272.46	52.51
r(AUUC)3 ds	1231.75	230.59
r(UA)6 ss	880.23	199.4
r(UA)6 ds	1340.73	231.92
d(GC)6 ds	444.22	297.65
d(GC)6 ss	21.99	24.13
r(GC)6 ds	3217.29	752.31
r(GC)6 ss	2031.26	270.45

* ss: single-stranded; ds: double-stranded. ** The K_d_ confidence interval is derived from the variance of the parameter fit. The reported values represent the mean K_d_ ± a 68% confidence range.

**Table 2:** Ligand–RBP7910 pairs and docking score standard deviation values for top five docking models (HDOCK). Predicted binding sites are in bold.

Ligand	Ligand Sequence	SD *
r(UUUU)3 ss	5′-GGUGGGUUA**UUUUUUUUUUUUU**AUCAACUGGG-3′	6.899
r(UUUU)3 ds	/Antisense	5.530
r(AUUU)4 ss	5′GGUGGGUUAU**AUUUAUUUAUUUAUUU**AUCAACUGGG-3′	7.470
r(AUUU)4 ds	/Antisense	7.107
r(AUUC)3 ss	5′-GGUGGGUUAU**AUUCAUUCAUUC**AUCAACUGGG-3′	6.530
r(AUUC)3 ds	/Antisense	4.917
d(GC)6 ss	5′-**CGCGCGCGCGCG**ATCAACTGGG-3′	6.249
d(GC)6 ds	/Antisense	16.677
r(GC)6 ss	5′-**CGCGCGCGCG**CGAUCAACUGGG-3′	12.336
r(GC)6 ds	/Antisense	3.523
r(UA)6 ss	5′-**UAUAUAUAUAUA**AUCAACUGGG-3′	10.156
r(UA)6 ds	/Antisense	3.392

* Docking score standard deviation across the top five docking models per ligand.

**Table 3:** Oligonucleotides designed for MST with predicted binding sites in bold.

Type of Oligos	Sequence
d(GC)6-Sense Cy5	5′-GGTGGGTTAT**CGCGCGCGCGCGA**TCAACTGGG-3′
d(GC)6-Antisense	5′-CCCAGTTGAT**CGCGCGCGCGCG**ATAACCCACC-3′
r(GC)6-Sense Cy5	5′-GGUGGGUUAU**CGCGCGCGCGCG**AUCAACUGGG-3′
r(GC)6-Antisense RNA	5′-CCCAGUUGAU**CGCGCGCGCGCG**AUAACCCACC-3′
r(UA)6-Sense Cy5	5′-GGUGGGUUAU**UAUAUAUAUAUA**AUCAACUGGG-3′
r(UA)6-Antisense	5′-CCCAGUUGAU**UAUAUAUAUAUA**AUAACCCACC-3′
r(AUUU)4-Sense Cy5	5′-GGUGGGUUAU**AUUUAUUUAUUUAUUU**AUCAACUGGG/3Cy5Sp/-3′
r(AUUU)4-Antisense	5′-CCCAGUUGAU**AAAUAAAUAAAUAAAU**AUAACCCACC-3′
r(AUUC)3-Sense Cy5	5′-GGUGGGUUAU**AUUCAUUCAUUC**AUCAACUGGG/3Cy5Sp/-3′
r(AUUC)3-Antisense	5′-CCCAGUUGAU**GAAUGAAUGAAU**AUAACCCACC-3′
r(UUUU)3-Sense Cy5	5′-GGUGGGUUA**UUUUUUUUUUUUU**AUCAACUGGG/3Cy5Sp/-3′
r(UUUU)3-Antisense	5′-CCCAGUUGAU**AAAAAAAAAAAAA**UAACCCACC-3′

## Data Availability

Data is contained within the article.
